# Supplementary material for: A Global Screen for Assembly State Changes of the Mitotic Proteome by SEC-SWATH-MS
Source: Cell Syst. 2020 Feb 26;10(2):133–155.e6. doi: 10.1016/j.cels.2020.01.001 (PMC7042714; doi:10.1016/j.cels.2020.01.001)

Q8WUX1 | S38A5_HUMAN | SLC38A5 JM24 SN2 SNAT5 PP7194
Monomer MW [kDa]: 51.457 Monomer expected elution fraction: 46

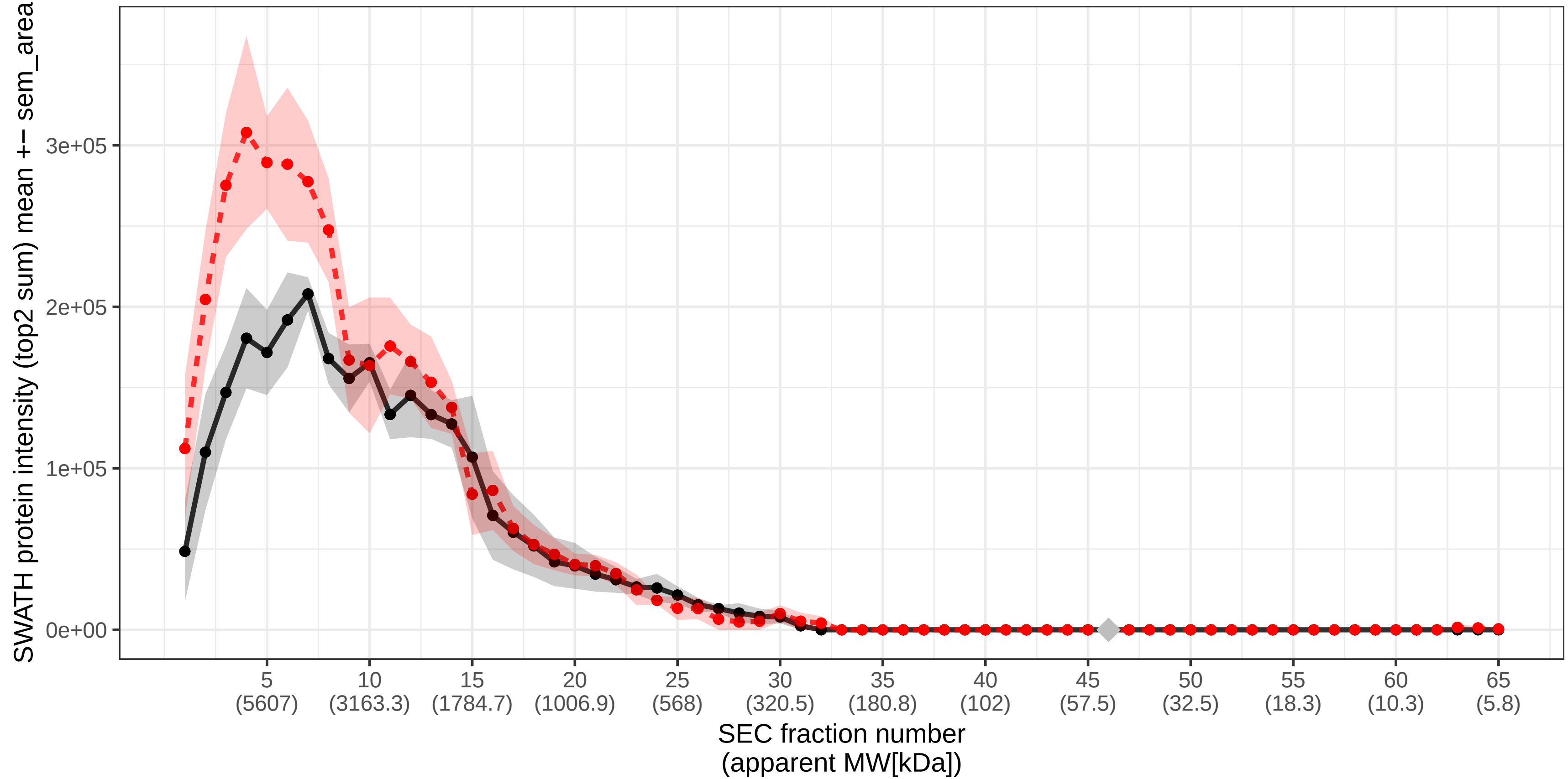

Supplement: Data S1. SEC-SWATH-MS Protein Chromatograms, Related to Figure 1 [file mmc6.zip › SECchrom_Q8WUX1_S38A5_HUMAN_SLC38A5_JM24_SN2_SNAT5_PP7194.pdf]
